# Supplementary material for: Identification and management of young infants with possible serious bacterial infection where referral was not feasible in rural Lucknow district of Uttar Pradesh, India: An implementation research
Source: PLoS One. 2020 Jun 4;15(6):e0234212. doi: 10.1371/journal.pone.0234212 (PMC7272098; doi:10.1371/journal.pone.0234212)
Supplement: S1 Table — (DOCX) [file pone.0234212.s001.docx]

**Supplementary Table 1: The public health infrastructure available in Lucknow district and in 4 Implementation Blocks**

| **Health Units** | **Lucknow District** | **4 Implementation blocks** |
| --- | --- | --- |
| Community Health Centers (CHC) | 09 | 04 |
| Primary Health Centers (PHC) | 33 | 16 |
| Sub-centers | 321 | 155 |
| Pediatricians at CHCs | 9 | 04 |
| Medical Officers at PHCs | 35 | 18 |
| Auxillary Nurse Midwives | 344 | 176 |
| Accredited Social Activists (ASHAs) | 1486 | 750 |
| ASHA Supervisors, Staff nurses/Lady Health  Visitor(Supervisory Staff) | 170 | 95 |
