# Supplementary material for: Identification and management of young infants with possible serious bacterial infection where referral was not feasible in rural Lucknow district of Uttar Pradesh, India: An implementation research
Source: PLoS One. 2020 Jun 4;15(6):e0234212. doi: 10.1371/journal.pone.0234212 (PMC7272098; doi:10.1371/journal.pone.0234212)
Supplement: S2 Table — (DOCX) [file pone.0234212.s002.docx]

**Supplementary Table 2: Actions taken over responses of family of sick infant in community/ PHC/CHC**

| If the family accepts referral for hospitalization- | - Arrangement of transport which is provided free of cost under *JSSK*^*^ - ASHA may accompany the baby to the nearest CHC/ SNCUs/ tertiary care hospital |
| --- | --- |
| If the family refuses referral for hospitalization BUT ready to visit health facility | - ASHA accompanies the baby to the nearest SC/PHC/CHC for diagnosis and treatment/ medical advice - ANM/Staff Nurse/ Medical officer assesses the young infant - Counselling of caregivers for urgent referral to CHC/ DH/ TCC (Tertiary care centre) - Arranged transport and explained whom to contact at the referral facility. - Provided Pre-referral dose – 1^st^ dose of each antibiotic |
| If the family refuses to accept hospital referral and prefers home treatment | - ASHA accompanied the PSBI case to the PHC/ANM for confirmation - ANM assessed the sick Young Infant, Proposes simplified treatment at SC/home - Family visits SC/PHC/CHC daily for simplified treatment dose - ASHA follows up the Young Infant daily at home till Treatment completion |
| If family refuses to attend even the first level health facility | - ASHA contacts the ANM and explains the situation. - Concerned ANM visits the sick Young Infant at home and informs Medical Officer - ANM followed the Sick Young Infant between 10-14 days for relapse or a new morbidity. - ASHA visited the house daily to observe the child till 14 days |

****JSSK-*** *Janani Shishu Suraksha Karyakaram* scheme provides free referral transport for new-born.

ASHA – Accredited Social Health Activist

ANM – Auxiliary Nurse Midwifery

SNCU – Sick Newborn Care Unit

CHC – Community Healthcare Centre

PHC – Primary healthcare Centre

SC – Sub-Centre
