# Supplementary material for: Identification and management of young infants with possible serious bacterial infection where referral was not feasible in rural Lucknow district of Uttar Pradesh, India: An implementation research
Source: PLoS One. 2020 Jun 4;15(6):e0234212. doi: 10.1371/journal.pone.0234212 (PMC7272098; doi:10.1371/journal.pone.0234212)
Supplement: S3 Table — (DOCX) [file pone.0234212.s003.docx]

**Supplementary Table 3: Initial training in four Intervention blocks**

| **Type of participants**  **[Timing of training]** | **Number of Batches** | **Number of participants**  **[Types]** | **Duration**  **(days)** | **Methods** | **Content of training** | **Training Material** |
| --- | --- | --- | --- | --- | --- | --- |
| **Training of Master trainers**  **[November 2016]** | 01 | 03  [national level training of project investigators] | 03 | Class room training - cases studies and videos.  Clinical practice sessions in the wards | Assessment, classification and treatment of sick young infant for very severe disease (PSBI), Pneumonia, local infection, feeding problem, and low birth weight | 1. Facilitator guidelines-*Management of the sick young infant of age up to 02 months* 2. Participant manual-*Management of the sick young infant of age up to 02 months* 3. Chart booklet 4. Photo booklet-Photograph for physicians-IMNCI   (*WHO-UNICEF*) |
| **Training of State Level Trainers**  **[April 2017]** | 01 | 24  (Pediatricians/MOs posted at CHCs, DH, and SNCUs) | 03 |  |  |  |
| **Training of Medical Officers**  **[May, Aug, Sept 2017]** | 04 | 11  [Medical officers of PHCs at their respective CHCs**]** | 03 | Classroom training using cases studies and videos and practical training at nearby SNCUs |  | 1. Participant manual 2. Chart booklet 3. Photo booklet-Photograph for physicians-IMNCI   (*WHO-UNICEF*) |
| **Training of ANMs and staff nurses at their respective CHCs**  **[ May 2017 ]** | 04 | 137  (20-30 participants/batch) | 03 |  | Same as for medical doctors plus skill development for giving Inj Gentamicin | 1. Participant manual 2. Chart booklet 3. Photo booklet 4. Photograph for physicians-IMNCI   (*WHO-UNICEF* |
| **Training of community Health workers(ASHA) and their supervisors**  **[June 2017]** | 14 | 700  (45-55/batch) | 01 | Classroom  Training using videos | Identification of danger signs in sick young infants and referral | **Chart booklet –**Management of the sick young infant (translated in Hindi)  **Picture booklet** |
