# Supplementary material for: Identification and management of young infants with possible serious bacterial infection where referral was not feasible in rural Lucknow district of Uttar Pradesh, India: An implementation research
Source: PLoS One. 2020 Jun 4;15(6):e0234212. doi: 10.1371/journal.pone.0234212 (PMC7272098; doi:10.1371/journal.pone.0234212)
Supplement: S4 Table — (DOCX) [file pone.0234212.s004.docx]

**Supplementary Table 4: Re-orientation training of ASHAs and ANMs in the intervention blocks (Nov 2017-Feb 2019)**

| **Meetings** | **Participants** | **Number of meetings** | **Activities are undertaken by Project team** | **Support from government officials** |
| --- | --- | --- | --- | --- |
| Monthly Cluster* meetings at Community Health centers | CHWs (ASHA) and ASHA *Sangini* | 215 meetings  (50-60 meetings per CHC) | - Reorientation of ASHAs on the identification of danger signs in young infants with focus on the seven of PSBI in a young infant - Handholding of ASHA to count RR and correct use of thermometer in a young infant - Discussion on problems faced by ASHA in the documentation of HBNC visit and handholding for maintenance of records - Experience sharing by ASHAs who identified a young infant with danger signs and successfully referred the case to a health facility | - Support was given by Health Education Officer (HEO) of the respective CHC to the project team in reorientation and handholding of ASHAs - Some Cluster meetings were also attended by the Medical superintendent of the CHC. |
| Weekly meetings of ANMs at CHCs | ANMs | 175 meetings  (40-50 meetings per CHCs) | - Reorientation of ANMs on assessment, classification and pre-referral treatment of sick young infant. - Identification of seven PSBI signs in a young infant - Motivation to administer Pre referral dose(PRD) before referral of a PSBI case to an Health Facility - Revision of Simplified treatment (inj. Gentamicin and distab/syp. Amoxicillin) using Job aids - Felicitation of ANMs by awarding “Appreciation Certificate” | Support by HEOs and Medical Superintendent in reorienting ANMs on assessment of Sick young infants and motivating them to administer PRD before referring sick young infant to the HF |

*Cluster is a group of 20 ASHAs headed by a supervisor known as ASHA supervisor. There are approximately 8-10 clusters in each block.
