# Supplementary material for: Identification and management of young infants with possible serious bacterial infection where referral was not feasible in rural Lucknow district of Uttar Pradesh, India: An implementation research
Source: PLoS One. 2020 Jun 4;15(6):e0234212. doi: 10.1371/journal.pone.0234212 (PMC7272098; doi:10.1371/journal.pone.0234212)
Supplement: Attachment — Submitted filename: PONE-D-20-05531_reviewer-comp.pdf [file pone.0234212.s005.pdf]

PLOS ONE

Identification and management of young infants with possible serious bacterial infection where referral was not feasible in rural Lucknow district of Uttar Pradesh, India: An implementation research.

--Manuscript Draft--

**Manuscript Number:**	PONE-D-20-05531
**Article Type:**	Research Article
**Full Title:**	Identification and management of young infants with possible serious bacterial infection where referral was not feasible in rural Lucknow district of Uttar Pradesh, India: An implementation research.
**Short Title:**	Simplified treatment for possible serious bacterial infection in young infants in India
**Corresponding Author:**	Shally Awasthi<br>King George's Medical University<br>Lucknow, Uttar Pradesh INDIA
**Keywords:**	Possible serious bacterial infection; simplified treatment; home based newborn care; young infants; implementation; India
**Abstract:**	Background: Based on World Health Organization guidelines, Government of India recommended management of possible serious bacterial infection (PSBI) in young infants up to two months of age on an outpatient basis where referral is not feasible. We implemented the guideline in program setting to increase access to treatment with high treatment success and low resultant mortality.<br>Methods: Implementation research was conducted in four rural blocks of Lucknow district in Uttar Pradesh, India. It included policy dialogues with the central and state government and district level officials. A Technical Support Unit was established. Thereafter, capacity building across all cadres of health workers in the implementation area was done for strengthening of home based newborn care (HBNC) program, skills enhancement for identification and management of PSBI, logistics management to ensure availability of necessary supplies, monitoring and evaluation as well as providing feedback. Data was collected by the research team.<br>Results: From June 2017 to February 2019 there were 24,448 live births in a population of 856106. We identified 1302 infants up to 2 months of age with any sign of PSBI leading to a coverage of 53% (1302/2445), assuming an incidence of 10%. However, in the establishment phase the coverage was 33%, while it was 85% in the implementation phase. Accredited social health activists (ASHAs)identified 81.2% (1058/1302) cases while rest were identified by families. ASHAs increased home visits within first 7 days of life in home based newborn care program from 74.3% (2781/3738) to 89.0% (3128/3513) and detection of cases of PSBI from 1.6% (45/2781) to 8.7% (275/3128) in the first and last quarter of the project, respectively. Of these 18.7% (244/1302) refused referral to government health system and 6.7% (88/1302) were treated in a hospital. Among cases of PSBI, there were 13.3% (173/1302) cases of fast breathing in young infant aged 7-59 days in whom referral was not needed. Of these 147 were treated by oral amoxicillin and 95.2% (140/147) were cured. Among those who needed referral, simplified treatment was given when referral was refused. There were 2.9% (37/1302) cases of fast breathing at ages of 0-6 days of which 34 were treated by simplified treatment with100% (34/34) cured;66.5% (866/1302) were cases of clinical severe infection of which 685 treated by simplified treatment with94.2% (645/685)cured and 09 died;17.3% (226/1302) cases of critical illness of which 93 were treated by simplified treatment, as a last resort, 72% (67/93) cured and 16 died. Among 255 cases who either did not seek formal treatment or sought it at private facilities, 96 died.<br>Conclusion: Simplified treatment for PSBI is feasible in public program settings in northern India with good cure rates. It required system strengthening and supportive supervision.
**Order of Authors:**	Shally Awasthi
	Naveen Kesarwani
	Raj Kumar Verma

Powered by Editorial Manager® and ProduXion Manager® from Aries Systems Corporation

	Girdhar Gopal Agarwal
	Luxmi Shanker Tewari
	Ravi Krishna Mishra
	Lalji Shukla
	Arun Kumar Raut
	Shamim Ahmad Qazi
	Samira Aboubaker
	Yasir Bin Nisar
	Rajiv Bahl
	Monika Agarwal
**Additional Information:**	
**Question**	**Response**
**Financial Disclosure**<br><br>Enter a financial disclosure statement that describes the sources of funding for the work included in this submission. Review the submission guidelines for detailed requirements. View published research articles from *PLOS ONE* for specific examples.<br><br>This statement is required for submission and **will appear in the published article** if the submission is accepted. Please make sure it is accurate.<br><br>**Unfunded studies**<br>Enter: *The author(s) received no specific funding for this work.*<br><br>**Funded studies**<br>Enter a statement with the following details:<br>• Initials of the authors who received each award<br>• Grant numbers awarded to each author<br>• The full name of each funder<br>• URL of each funder website<br>• Did the sponsors or funders play any role in the study design, data collection and analysis, decision to publish, or preparation of the manuscript?<br>• **NO** - Include this sentence at the end of your statement: *The funders had no role in study design, data collection and analysis, decision to publish, or preparation of the manuscript.*<br>• **YES** - Specify the role(s) played.	The study was supported by World Health Organization Geneva, Switzerland (<br>World Health Organization: WHO<br>www.who.int) via Project ID: MCA00415. The funders had role in study conceptualization and review and editing of the manuscript.

* typeset	
**Competing Interests**<br><br>Use the instructions below to enter a competing interest statement for this submission. On behalf of all authors, disclose any competing interests that could be perceived to bias this work—acknowledging all financial support and any other relevant financial or non-financial competing interests.<br><br>This statement **will appear in the published article** if the submission is accepted. Please make sure it is accurate. View published research articles from *PLOS ONE* for specific examples.<br><br>**NO authors have competing interests**<br><br>Enter: *The authors have declared that no competing interests exist.*<br><br>**Authors with competing interests**<br><br>Enter competing interest details beginning with this statement:<br><br>*I have read the journal's policy and the authors of this manuscript have the following competing interests: [insert competing interests here]*<br><br>* typeset	The authors have declared that no competing interests exist.
**Ethics Statement**<br><br>Enter an ethics statement for this submission. This statement is required if the study involved:<br><br>• Human participants<br>• Human specimens or tissue<br>• Vertebrate animals or cephalopods<br>• Vertebrate embryos or tissues<br>• Field research<br><br>Write "N/A" if the submission does not require an ethics statement.	The study was approved by the Institutional Ethics committee of King George Medical University, Lucknow, Uttar Pradesh, India (vide 79th ECMH-A/P4) and Ethics Review Committee of World Health Organization, Geneva.

General guidance is provided below. Consult the submission guidelines for detailed instructions. **Make sure that all information entered here is included in the Methods section of the manuscript.**<br><br>**Format for specific study types**<br><br>**Human Subject Research (involving human participants and/or tissue)**<br>• Give the name of the institutional review board or ethics committee that approved the study<br>• Include the approval number and/or a statement indicating approval of this research<br>• Indicate the form of consent obtained (written/oral) or the reason that consent was not obtained (e.g. the data were analyzed anonymously)<br><br>**Animal Research (involving vertebrate animals, embryos or tissues)**<br>• Provide the name of the Institutional Animal Care and Use Committee (IACUC) or other relevant ethics board that reviewed the study protocol, and indicate whether they approved this research or granted a formal waiver of ethical approval<br>• Include an approval number if one was obtained<br>• If the study involved *non-human primates*, add *additional details* about animal welfare and steps taken to ameliorate suffering<br>• If anesthesia, euthanasia, or any kind of animal sacrifice is part of the study, include briefly which substances and/or methods were applied<br><br>**Field Research**<br><br>Include the following details if this study involves the collection of plant, animal, or other materials from a natural setting:<br>• Field permit number<br>• Name of the institution or relevant body that granted permission	
**Data Availability**<br><br>Authors are required to make all data underlying the findings described fully	No - some restrictions will apply

available, without restriction, and from the time of publication. PLOS allows rare exceptions to address legal and ethical concerns. See the PLOS Data Policy and FAQ for detailed information.<br><br>A Data Availability Statement describing where the data can be found is required at submission. Your answers to this question constitute the Data Availability Statement and **will be published in the article**, if accepted.<br><br>**Important:** Stating 'data available on request from the author' is not sufficient. If your data are only available upon request, select 'No' for the first question and explain your exceptional situation in the text box.<br><br>Do the authors confirm that all data underlying the findings described in their manuscript are fully available without restriction?	
**Describe where the data may be found in full sentences. If you are copying our sample text, replace any instances of XXX with the appropriate details.**<br><br>• If the data are **held or will be held in a public repository**, include URLs, accession numbers or DOIs. If this information will only be available after acceptance, indicate this by ticking the box below. For example: *All XXX files are available from the XXX database (accession number(s) XXX, XXX.*).<br>• If the data are all contained **within the manuscript and/or Supporting Information files**, enter the following: *All relevant data are within the manuscript and its Supporting Information files.*<br>• If neither of these applies but you are able to provide **details of access elsewhere**, with or without limitations, please do so. For example:<br><br>*Data cannot be shared publicly because of [XXX]. Data are available from the XXX Institutional Data Access / Ethics*	All relevant data are within the manuscript and its Supporting Information files.

Powered by Editorial Manager® and ProduXion Manager® from Aries Systems Corporation

Committee (contact via XXX) for researchers who meet the criteria for access to confidential data.<br><br>*The data underlying the results presented in the study are available from (include the name of the third party and contact information or URL).*<br>• This text is appropriate if the data are owned by a third party and authors do not have permission to share the data.<br><br>* typeset	
Additional data availability information:	Tick here if the URLs/accession numbers/DOIs will be available only after acceptance of the manuscript for publication so that we can ensure their inclusion before publication.

Powered by Editorial Manager® and ProduXion Manager® from Aries Systems Corporation

Manuscript

Click here to download Manuscript 1_Manuscript SA 26.02.2020.docx

Title: Identification and management of young infants with possible serious bacterial infection where referral was not feasible in rural Lucknow district of Uttar Pradesh, India: An implementation research.

Shally Awasthi[1*], Naveen Kesarwani[1¶], Raj Kumar Verma[1¶], Girdhar Gopal Agarwal[2], Luxmi Shanker Tewari[1], Ravi Krishna Mishra[1],Lalji Shukla[1], Arun Kumar Raut[1],Shamim Ahmad Qazi[3], Samira Aboubaker[3], Yasir Bin Nisar[4], RajivBahl[4], Monika Agarwal[5]

[1]Department of Pediatrics, King George’s Medical University, Lucknow, Uttar Pradesh, India

[2]Department of Statistics, University of Lucknow, Lucknow Uttar Pradesh, India

[3]Department of Maternal Newborn Child and Adolescent Health, World Health Organization, Geneva, Switzerland(retired)

[4]Department of Maternal Newborn Child and Adolescent Health and Aging, World Health Organization, Geneva, Switzerland

[5]Department of Social and Preventive Medicine, King George’s Medical University, Lucknow Uttar Pradesh, India

*Corresponding Author

E-mail: shally07@gmail.com

¶ These authors contributed equally to this work

Abstract

Background: Based on World Health Organization guidelines, Government of India recommended management of possible serious bacterial infection (PSBI) in young infants up to two months of age on an outpatient basis where referral is not feasible. We implemented the guideline in program setting to increase access to treatment with high treatment success and low resultant mortality.

Methods: Implementation research was conducted in four rural blocks of Lucknow district in Uttar Pradesh, India. It included policy dialogues with the central and state government and district level officials. A Technical Support Unit was established. Thereafter, capacity building across all cadres of health workers in the implementation area was done for strengthening of home based newborn care (HBNC) program, skills enhancement for identification and management of PSBI, logistics management to ensure availability of necessary supplies, monitoring and evaluation as well as providing feedback. Data was collected by the research team.

Results: From June 2017 to February 2019 there were 24,448 live births in a population of 856106. We identified 1302 infants up to 2 months of age with any sign of PSBI leading to a coverage of 53% (1302/2445), assuming an incidence of 10%. However, in the establishment phase the coverage was 33%, while it was 85% in the implementation phase. Accredited social health activists (ASHAs)identified 81.2% (1058/1302) cases while rest were identified by families. ASHAs increased home visits within first 7 days of life in home based newborn care program from 74.3% (2781/3738) to 89.0% (3128/3513) and detection of cases of PSBI from 1.6% (45/2781) to 8.7% (275/3128) in the first and last quarter of the project, respectively. Of these 18.7% (244/1302) refused referral to government health system and 6.7% (88/1302) were treated in a hospital. Among cases of PSBI, there were 13.3%

(173/1302) cases of fast breathing in young infant aged 7-59 days in whom referral was not needed. Of these 147 were treated by oral amoxicillin and 95.2% (140/147) were cured. Among those who needed referral, simplified treatment was given when referral was refused. There were 2.9% (37/1302) cases of fast breathing at ages of 0-6 days of which 34 were treated by simplified treatment with100% (34/34) cured;66.5% (866/1302) were cases of clinical severe infection of which 685 treated by simplified treatment with94.2% (645/685)cured and 09 died;17.3% (226/1302) cases of critical illness of which 93 were treated by simplified treatment, as a last resort, 72% (67/93) cured and 16 died. Among 255 cases who either did not seek formal treatment or sought it at private facilities, 96 died.

Conclusion: Simplified treatment for PSBI is feasible in public program settings in northern India with good cure rates. It required system strengthening and supportive supervision.

Keywords: Possible serious bacterial infection, simplified treatment, home based newborn care, young infants, implementation, India.

Introduction

India has been struggling tenaciously to tackle morbidity and mortality in children. Infant mortality rate in India is 33 versus 41 per 1000 live births in Uttar Pradesh the most populous state, whereas neonatal mortality rate is 23 versus 30 per 1000 live births [1]. Leading infectious causes of mortality among young infants are pneumonia, sepsis and meningitis [2, 3]. With an estimated 9.8% percent case fatality risk of possible serious bacterial infection (PSBI) [4] in low resource setting, survivors are also at risk of long term disability [5].Therefore, early diagnosis and treatment is crucial for saving lives of young infants.

Early identification of illness by caregivers in sick young infants is difficult due to non-specific signs. World Health organization (WHO) recommends postnatal home visits by healthcare providers to facilitate early identification of danger signs and promote care seeking [6]. Government of India launched the Home Based Newborn Care (HBNC) program in 2011 for accelerated reduction of neonatal mortality, especially in rural and remote areas where access to health care is challenging [7]. In the HBNC program, an Accredited Social Health Activist (ASHA) visits mothers and neonates at home at least six times within the first 42 days of birth and refer sick infants to public health facility. WHO recommends sick young infants with PSBI be referred to a hospital for inpatient treatment with a seven to ten-day course of injection ampicillin or benzyl penicillin plus gentamicin [8, 9]. But hospitalization and life-saving treatment may not be accessible, acceptable or affordable to families in settings with high new-born mortality [10, 11, 12, 13].

In 2014, Ministry of Health and Family Welfare, Government of India issued guideline for Auxiliary Nurse and Midwife (ANMs)for management of sepsis in young infants where referral is not feasible i.e., use of injection gentamicin plus oral amoxicillin as a pre-referral dose or completion of treatment for 7 days [14].WHO, in 2015, published the guideline for

management of PSBI in young infants when referral is not feasible [15] based on evidence from several countries [13, 16, 17, 18, 19], which recommended a simplified treatment regimen with oral amoxicillin plus injection gentamicin for treatment of two sub-categories of PSBI. In 2017, government of India issued an addendum with a revised list of signs for classifying young infants as PSBI, consistent with the WHO guideline, removing nasal flaring, grunting, 10 or more skin pustules and blood in stool and recommended treatment of fast breathing young infants 7-59 day of age with oral amoxicillin only [20].

The primary objective of this operational research was to evaluate acceptability, effectiveness and scalability of simplified treatment regimen to cases of PSBI when referral is not accepted in a HBNC program setting in Uttar Pradesh. This implementation research was also conducted in three other states of India- Haryana, Himachal Pradesh and Maharashtra.

Study Methodology

Demographic Details

Lucknow district in the Uttar Pradesh state, India is located at an elevation of 123 meters above sea level, has a crude birth rate of Uttar Pradesh is 25.9 per 1000 population [1].Female literacy rate is 79.4% in women in 15-49 years' group. Lucknow district has an estimated population of 4,589,838 of which 1,550,942(33.8%) live in the eight rural blocks [21].Implementation research activities were conducted in four of the eight rural blocks, namely Mall, Gosaiganj, Sarojini Nagar and Kakori, whose population is856106. [22]

Health infrastructure

Each village has an ASHA, a voluntary community-based female worker with at least eight years of formal education [23]. She works as a link between the community and public health facilities, covers a population of about approximately1000, an average village size. A sub-center (SC) has one ANM for a population of approximately 5000 to 8000or 5 – 7 villages [24]. For every 6 SCs there is one Primary Health Center (PHC) covering a population of 30,000 with outpatient and in-patient facility of 6 beds and one delivery room. Each PHC has 1-2 medical doctor (either trained in modern medicine with MBBS degree and/or in alternative systems of medicine such as ayurveda, yoga therapy, unani and siddh medicine and homeopathy together called as AYUSH, three nurses, one ANM and a pharmacist [25].For 4 PHCs there is a Community Health Center (CHC) covering a population of80,000 to 1,20,000 with outpatient and in-patient facility with 30 beds, a delivery room, operation theater, one sick newborn stabilizing unit with phototherapy and warmer units and a kangaroo mother care unit with beds for mothers with babies born with birth weight of less than 1800 g [26].CHC has one or two general doctors, few specialists one each being general physician, pediatrician, obstetrician, surgeon and anesthesiologist, seven nurses, one ANM, one public health midwife and one pharmacist. There is also facility for essential bed side laboratory investigations. All CHCs have government ambulance services for transport of patients, including pregnant women and young infants and are designated as the first referral units. Available public health infrastructure in Lucknow district and four implementation blocks is shown in **S1 Table**.

ASHA and HBNC Program: In each village, HBNC program is being implemented with ASHA as its grass root functionary. In this program, an ASHA does home visits on specific days (Days 1, 3, 7, 14, 21, 28 and 42)with day 1 visit only for mothers who delivered at home. During each visit, in addition to post-natal care of mothers they perform various

activities to ensure well-being of young infants such as physical examination, temperature and weight recording, respiratory rate counting and counselling mothers for exclusive breast feeding etc. ASHA counsels mothers and looks for signs of illness in young infants including diarrhea and dehydration, jaundice, skin infections and PSBI. [7]

ASHA-*Sangini* (Supervisors):All ASHA workers of a block are grouped in clusters of 20, each headed by an ASHA-*Sangini* who is responsible for supportive supervision and hand-holding in the field. Cluster meetings are organized monthly at the CHC for reporting routine activities to the manager and for ongoing skills enhancement.

ANM:ANMs are responsible for routine immunization at SC/PHC/CHC and administration of certain drugs/ treatment approved to community at SC. [24]They maintain records of pregnant women for ante-natal, deliveries, post-natal care and immunization of children in their area. All ANM in a block meet weekly and report their activities to the CHC medical officers and collect their supplies.

Identification of cases of PSBI: During routine HBNC visits or additional visits, ASHA identifies sick young infants including those with PSBI signs. ASHAs advise parents of all such infants to visit the CHC/PHC/tertiary care hospitals for medical care. ASHA can consult the ANM, if she is available in the village on that particular day. When ANM confirms a case of PSBI, they are referred to a public hospital/ tertiary care facility after administration of pre-referral dose of injection gentamicin and oral amoxicillin. See **S2 Table**. The exception is for only fast breathing (> 60 breaths per minute) in 7-59 days old infants who are given 7 days of oral amoxicillin without referral. Similar process is carried out by attending physician for young infants with PSBI signs who present at a PHC/CHC. If referral is not accepted, then the sick infant is categorized into critical illness (convulsions, no movement on stimulation, not feeding at all) or clinical severe infection (less than normal

movements or movement only on stimulation, not feeding well, severe chest indrawing, temperature of >= 38°Cor <35.5^{o}C) or severe pneumonia (fast breathing 0-6 days). WHO recommended simplified treatment is offered for clinical severe infection and severe pneumonia and referral is reinforced in those with critical illness as they are unable to take oral antibiotics [15].

Simplified Treatment: Oral amoxicillin twice a day (40-50 mg/dose) plus injection gentamic in once a day (7 mg/Kg) for 7 days was administered to those with clinical severe infection or severe pneumonia [14]. Parents either brought the case daily to the health facility to receive injectable gentamicin or ANM visited home of the case daily for administering it. Parents gave the first dose of oral amoxicillin under medical supervision and then subsequent doses at home. ASHA visited such cases almost daily to ensure adherence to treatment. Young infants aged 7-59 days with only fast breathing were given only oral amoxicillin twice a day for 7 days [14]. Cases were categorized as “treatment success” when the care providers perceived that the infant was cured or better and as “treatment failure” when condition worsened or there were persistent signs on day 8 or the patient died.

Policy Dialogue: For informing policy makers and for their approval for implementation research work, national as well as state level meetings were held prior to initiation of the project as the implementation research was done by the state government and health care functionaries. There was a continuous communication of the investigators with the implementers and the policy makers.

<u>National level meeting</u>: There were two meetings held at national level. First meeting was held in October 2016. It was attended by National advisors to government, representatives from Central Ministry of Health and family welfare, investigators of the four participating states, the World Health Organization representatives and expert advisors from Save the

Children. Outcome of the meeting was adaptation of guidelines for PSBI management in young infants where hospitalization is not feasible [14]. The second meeting was held with all the stakeholders who were present in the first meeting in New Delhi in July 2017. During this meeting, plans of all the four research sites were reviewed and discussed. Suggestions and recommendations to and from the government were incorporated customized to every research site.

State level meeting: A meeting of investigators with the Director, managing director and general manager Child health of Uttar Pradesh-National Health Mission was held ~~on~~ January 2017. They were briefed about the implementation research. All the officials appreciated and supported it and assured the availability of Sick newborn care unit and Kangaroo Mother Care units in four implementation blocks for management of the referred sick young infants. The director proposed incentives of INR 100 to the ASHA workers on successful referral of cases of PSBI to public health facility. He also recommended a brief re-training, especially for ASHA workers on management of PSBI. A technical advisory group (TAG)was formed to support and guide the research. Its members were representatives of state government and multilateral agencies working in child health such as UNICEF and Save the Children. Functions of the TAG was to review program implementation once in 6 months and provide inputs. At the end of the implementation phase, a state level dissemination meeting was also held where members of TAG were invited.

Role of technical Support unit (TSU): A TSU was established at KGMU which had the investigators and research staff trained by the investigators for supportive supervision of ASHAs and ANMs; effective implementation of HBNC program and simplified treatment for PSBI when referral was not feasible; necessary data collection from health care providers' records for continuous evaluation; and provide feedback to the program for quality improvement and for assessing effectiveness of the implementation research.

Role of district health staff: Government doctors conducted trainings for strengthening home based newborn care program and giving simplified treatment, logistic support and ensured supplies as well as continued their routine monitoring and supervisions. ASHA and ANMs conducted their routine activities which now included identification and management of cases of PSBI by simplified treatment where referral was refused.

Ethical Approval

The study was approved by the Institutional Ethics committee of King George Medical University, Lucknow, Uttar Pradesh and Ethics Review Committee of World Health Organization, Geneva.

Phases of the project

The project had two distinct phases: Establishment phase and Implementation phase.

Establishment phase (June 2017 to Feb 2018): The activities during this phase were:

a. **Training:** Initial training of master trainers for PSBI was conducted centrally in New Delhi by the WHO facilitators using pre-validated materials [15, 27]. The master trainers trained pediatricians and some medical officers in the state public health system for them to become the trainers for medical officers, nurses, ANMs, ASHAs and ASHA "*Sangini*". The training emphasized on the acquisition of necessary skills on identification and management of PSBI or neonatal sepsis in young infants (**S3 Table**).The research staff hired were trained by project investigators on HBNC and PSBI over three days, using the modules provided by the WHO which were used for the training for trainers. In addition, they were taken to the neonatal intensive care unit and sick newborn care unit of King George's Medical University and shown all signs of PSBI at least twice over a period of one week by the project investigator. There were multiple transfers of doctors and ANMs during the project. One to one training of new doctors and ANMs with refresher training

of those trained earlier was conducted by TSU staff at their place of posting, which mandated by the government that they must remain in their places of duty all day. Newly inducted ASHA were also trained at CHCs (**S3 Table).**

b. **Baseline survey:** This survey was conducted in 120 villages of four implementation rural blocks of Lucknow using the thirty cluster sampling technique. In each block, 30 villages were included based on the population enumerated in census 2011[1]. The villages were selected on the basis of size as small villages (<200 households), medium sized villages (200-400 households) and large sized villages (>400 households) in the ratio of 4:3:1 respectively. Mothers of infants 0-59 days was the unit if this survey. Mothers of all the young infants (0-59 days' age) registered with the ASHA in selected villages were visited and interviewed after taking informed written consent. Using a predesigned, structured questionnaire they were asked about the number of HBNC visits done for their young infant and what the ASHA did on each visit. ASHAs of selected villages were interviewed using a predesigned, structured questionnaire and in addition one ASHA who was doing HBNC on the day of survey was interviewed and observed as she conducted the procedures laid down in HBNC. All the ASHA were interviewed to assess training given to them and their knowledge about HBNC and the functional status of HBNC kit given to them was evaluated. A predesigned, structured interview questionnaire was used.

During this survey, to assess the knowledge of HBNC, recognition and management of PSBI, in-depth qualitative interview of a few mothers, ANMs and doctors was also done by purposive sampling. Baseline audit of health facilities was done to check for availability of oral amoxicillin, injectable gentamicin, 1 ml syringes and gentian violet lotion.

c. **Data Collection for process indicators:** From the records maintained by the district health staff, data was abstracted by TSU on numbers of pregnant women and deliveries

per village, number of times HBNC visit was done for each infant with findings of each visit like weight, respiratory rate, temperature and presence of any danger signs or signs of PSBI. In about 15% of such visits the TSU staff observed the practice of ASHA workers and identified areas which needed strengthening either with support of the ASHA-"*sangini*" or through re-training. The district health continued usual independent monitoring and evaluation of the HBNC program.

The TSU staff also collected information about cases of PSBI with, their presenting symptoms from the records maintained by the district health staff. Each PSBI case was visited by the research staff to validate findings abstracted as well as collect information on place of treatment, type of treatment specifically simplified treatment when referral was not accepted, response to administered treatment on days 4 and 8. Data on cases of PSBI on simplified treatment was collected by the research staff but in cases where parents took their child to a private provider or accepted referral to a district hospital, follow-up at home could not be done.

d. **Refresher training and support:** Members of TSU attended routine monthly cluster meetings of ASHAs and ANMs in their respective blocks. During this time, they scheduled refresher training on HBNC and PSBI program for ASHA with medical officers or ASHA "*sangini*" and observed them. During such trainings, skills which were found to be deficient like respiratory rate counting, physical examination to identify signs of infection and counselling for referral were re-enforced. ANMs were motivated by medical officers to administer pre-referral dose and in providing treatment at home/SC. Experiences on successful case identification and/or referral were also shared to encourage others. Medical officers appreciated ASHA who identified infants with danger signs and referred them for management. These meetings were also utilized for dialogue and fostering linkages between ASHA and their respective ANMs. See **S4 Table**

e. **Management Information System (MIS):** Common case report forms were developed and customized in India for all the sites for research data collection. It included five forms: 1) Pregnancy identification and listing form, 2) Postnatal Home visit record, 3) Sick Young infant assessment form, 4) Facility Follow-up form (for record of Simplified Treatment given), 5) Home follow up and outcome. Information collected was entered in the secure web based application, Research Electronic Data Capture “RED Cap”[28]. Information collected from the field was checked and verified before entry into the database. Data analysis was done using MS excel and SPSS version 18.0[29] Univariate distribution of variables was done and presented as proportion with 95% confidence interval where required. Since it was an implementation research no inferential statistical analysis was done.

The establishment phase took around 9 months to stabilize and deliver optimal performance with reference to number of cases of sick infants identified and administering of pre-referral dose and/or simplified treatment.

Implementation Phase (Mar 2018 to Feb 2019)

After the establishment phase, the intervention activities continued in the implementation mode, performing same activities as done in the establishment phase. The only new activity done during this phase was midline survey.

f. **Midline Survey:** One year after baseline survey, another survey was done using the same methodology as baseline survey. Same villages were visited as done in baseline survey however, mothers of infants ≤2 months’ age were new. If there was no mother in the village selected earlier, adjoining village of the same size was visited. During this survey, interview of mothers was done to evaluate the change in knowledge level. ASHA interview and observation was also done to evaluate the change in her skills. Interview of medical officers and ANMs was done to evaluate the change in their knowledge for

treatment of young infants and also to have a feedback for the simplified treatment of PSBI.

Results

Study Characteristics

At the initiation of the research, during baseline survey, structured interviews of 118 ASHAs and in-depth interview of 40 mothers with young infants. It was found that in the implementation blocks of Lucknow district, as in rest of the state of Uttar Pradesh, HBNC training had been given to ASHA workers at the time of recruitment. This training had a larger knowledge component and relatively small skills development component. There was no in-service reinforcement of training. Mothers informed that during the HBNC visits that ASHAs mostly weighed the young infants and inquiring about their wellbeing, without physical examination of the child. ASHA seldom measured axillary temperature or respiratory rate. On observation ASHAs were unable to use the respiratory rate counter as they either could not operate it or the battery had discharged. Thermometers were non-functional in 19 %(21/111) of HBNC kits.

In-depth interviews of 24 physicians and 79 ANMs was done during midline survey. They were asked that before this implementation research how would they manage a sick young infant and whether they had given injectable antibiotics to such cases. At the level of PHC/CHC the medical doctors replied that they would immediately refer a “very” sick young infant who was not feeding well or refusing feeds, not moving or moving less or convulsion or had jaundice to a higher center if there was no pediatrician available at that point of time. No pre-referral antibiotics or advise for maintaining temperature and feed during transportation was given at PHC/CHC. Doctors treated “minor” ailments in young infants

such as local skin infection was with topical and/or oral antibiotics, diarrhea in a case accepting feeds well with oral rehydration solution, and fever with paracetamol. The doctors as well as ANMs were hesitant in administering injectable antibiotics to sick young infants, even though they were competent in given injectable vaccines.

Baseline health facility audit showed that there was shortage of oral amoxicillin suspension in most facilities and that amoxicillin dispersible tablet was not available. Vials of gentamicin injections were available in the pharmacy of all health facilities but not issued to the ANMs for use in the field on sick young infants. One ml injections with 26 gauze syringes were not present in the government supply.

This implementation research was conducted in four rural blocks of Lucknow district with a population of 856,106. During the study period from 01 June 2017 to 28 Feb 2019, there were 25028 deliveries, of which 24448 were live births. ASHAs visited 20316 newborns **(Table 1).**

Table 1: Study Population parameters and Home Based Newborn Care visits from 1st Jun 2017 to 28th Feb 2019

<table>
<tr><th colspan="3">Parameters</th><th>Number</th></tr>
<tr><td colspan="3">Population of catchment area (Census 2011)</td><td>856106</td></tr>
<tr><td colspan="3">Expected number of deliveries in the catchment area*</td><td>22174</td></tr>
<tr><td colspan="3">Actual number of deliveries in the catchment area (Live Births + Still Births)</td><td>25028</td></tr>
<tr><td colspan="3">Total live births identified</td><td>24448</td></tr>
<tr><th colspan="4">Home Based New Born care visits by ASHA (From June 2017 to Feb 2019)</th></tr>
<tr><th></th><th>Eligible</th><th>Visited</th><th>%</th></tr>
<tr><td>Day 1 (for home deliveries only)</td><td>1325</td><td>840</td><td>63.4</td></tr>
<tr><td>Postnatal visits by Day 3</td><td>24448</td><td>14838</td><td>60.7</td></tr>
<tr><td>Postnatal visits by Day 7</td><td>24448</td><td>18106</td><td>74.1</td></tr>
<tr><td>Postnatal visits by Day 14</td><td>24199</td><td>18296</td><td>75.6</td></tr>
<tr><td>Postnatal visits by Day 21</td><td>23950</td><td>18183</td><td>75.9</td></tr>
<tr><td>Postnatal visits by Day 28</td><td>23453</td><td>17434</td><td>74.3</td></tr>
<tr><td>Postnatal visits by Day 42</td><td>22179</td><td>17844</td><td>80.4</td></tr>
</table>

*As per Crude Birth Rate by Census 2011 (1)

In a population of 856106, 24,448 live births were recorded from June 2017 to February 2019. Based on 10% incidence rate of PSBI, 2445 infants up to 2 months of age were expected to have any sign of PSBI. We identified 1302 infants up to 2 months of age with any sign of PSBI leading to a coverage of 53% (1302/2445). However, in the establishment phase the coverage was 33%, while it was 85% in the implementation phase.

As a result of supportive supervision of the ASHAs in the HBNC program, identification of PSBI and fast breathing cases improved in each successive quarter (**Table 2).**

Table 2: Identification and management of Possible serious bacterial infection cases by establishment and implementation Phases

	**Phases I: Establishment (Jun17- Feb18)**			**Phase II: Implementation (Mar18-Feb19)**			
	**Quarter 1** (Jun17-Aug17)	**Quarter 2** (Sep17-Nov17)	**Quarter 3** (Dec17-Feb18)	**Quarter 1** (Mar18-May18)	**Quarter 2** (Jun18-Aug18)	**Quarter 3** (Sep18-Nov18)	**Quarter 4** (Dec18-Feb19)
Live births	3738	3691	3124	3140	3409	3833	3513
Live births visited by ASHA in 1st week of life n, (%)	2781, (74.3%)	2950, (79.9%)	2545, (81.4%)	2618, (83.3%)	2918, (85.5%)	3376, (87.8%)	3128, (89.0%)
PSBI Identified n, (%)	45/ 2781, (1.6%)	127/2950, (4.4%)	106/2545, (4.1%)	261/2618, (9.9%)	239/2918, (8.1%)	249/3376, (7.3%)	275/3128, (8.7%)
Fast Breathing on at 7-59 days of age n, (%)	-	17/127, (13.3%)	30/106, (28.3%)	39/261, (14.9%)	22/239, (9.2%)	46/249, (18.4%)	56/275, (20.3%)
Simplified treatment n, (%)	30/127, (66.7%)	100/127, (78.7%)	81/106, (76.5%)	195/261, (74.7%)	170/239, (71.2%)	188/249, (75.5%)	195/275, (70.9%)
Pre-referral Dose of injection gentamicin	0/2	1/5	1/1	0/3	4/7	4/5	6/10
Refused referral and accepted simplified treatment	30/45, (66.7%)	100/127, (78.7%)	81/106, (76.5%)	195/261, (74.7%)	170/239, (71.2%)	188/249, (75.5%)	195/275, (70.9%)
Accepted referral and went to tertiary care hospital	05/45 (11.1%)	09/127 (7.0%)	01/106 (0.9%)	09/261 (3.4%)	24/239 (10.0%)	16/249 (6.4%)	24/275 (8.7%)
Refused referral and treatment at	10/45 (22.2%)	18/127, (14.2%)	24/106, (22.7%)	57/261, (21.9%)	45/239, (18.9%)	45/249, (18.1%)	56/275, (20.3%)

public health facility							

Abbreviation: PSBI – Possible serious bacterial infection

The proportion of identified PSBI increased from 1.6% in firstquarter to 8.7% in the last quarter.

Of the 1302 cases of PSBI, 1058 (81.3%) were identified by ASHA and 244 (18.7%) by families **(Fig 1).**Of 1003 brought to a PHC/CHC, 812 refused referral to a hospital but accepted simplified antibiotic treatment. Eighty-eight young infants (33 accepting referral to a hospital from PHC/CHC and 55 brought directly by families) were treated at a district hospital. Parents of 58 (4.5%)young infants took either used home remedies, sought care from faith healers or didn't receive any formal treatment, whereas parents of 197 (15.2%) young infants sought treatment from private practitioners (11 initially went to government facility but refused treatment there).

Fig1. Places of Identification and Management of cases of Possible Serious Bacterial Infections.

Details of management and outcomes by sub-classification of illness is given in **Table 3.**

Table 3: Identification, classification & outcome of treatment in infants (0-59 days) with Possible Serious Bacterial Infection

**Parameters**	**All PSBI cases**	**Fast Breathing 7-59 days**	**Fast Breathing 0-6 days**	**Clinical Severe Infection 0-59 days**	**Critical Illness 0-59 days**
A. **Identification and Referral History**	**N=1302 n/m (%)**	**N=173 n/m (%)**	**N=37 n/m (%)**	**N=866 n/m (%)**	**N=226 n/m (%)**
**Identified by Families and brought to Sub-centre/ Primary Health Centre/ Community Health Centre**	189/1302 (14.5)	21/173 (12.1)	18/37 (48.6)	103/866 (11.9)	47/226 (20.8)
**Identified by families and brought to District Hospital / Teaching Hospital**	55/1302 (4.2)	1/173 (0.6)	0/37 (0)	38/866 (4.4)	16/226 (7.1)
**Identified by ASHA**	1058/1302 (81.3)	151/173 (87.3)	19/37 (45.9)	725/866 (83.7)	163/226 (31.4)
a) Went to Subcentre/ Primary Health Centre/ Community	814/1058 (76.9)	127/151 (84.1)	17/19 (89.5)	599/725 (82.6)	71/163 (43.6)

Health Centre					
b) Refused referral to government health system	244/1058 (23.1)	24/151 (15.9)	2/19 (10.5)	126/725 (17.4)	92/163 (56.4)
i)Went to Private for treatment	186/244 (76.2)	24/24 (100)	2/2 (100)	110/126 (87.3)	50/92 (54.3)
ii)No formal treatment received*	58/244 (23.8)	0/24 (0)	0/0 (0)	16/126 (12.7)	42/92 (45.7)
**Total infants assessed at Subcentre/ Primary Health Centre/ Community Health Centre**	1003/1302 (77.0)	148/173 (85.5)	35/37 (94.6)	702/866 (81.1)	118/226 (52.2)
Referred to District Hospital	33/1003 (3.2)	0/148 (0)	1/35 (2.9)	12/702 (1.7)	20/118 (17)
Treated as outpatient on simplified treatment that does not need referral	147/1003 (14.7)	147/148 (99.3)	-	-	-
Refused referral to a hospital and accepted simplified treatment	812/1003 (81)	-	34/35 (97.1)	685/702 (97.6)	93/118 (78.8)
Refused treatment at Primary Health Centre/ Community Health Centre	11/1003 (1.1)	1/148 (0.7)	0/0 (0)	5/702 (0.7)	5/118 (4.2)
B. **Follow up**	**N=959 n/m (%)**	**N=147 n/m (%)**	**N=34 n/m (%)**	**N=685 n/m (%)**	**N=93 n/m (%)**
**Infants followed up on day 4 (*mandatory day as to WHO guideline*)**	919/959 (95.8)	146/147 (99.3)	34/34 (100)	663/685 (96.8)	76/93 (81.7)
Infants completed all follow-up visits on Day 4 and Day 8	898/959 (93.6)	142/147 (96.6)	34/34 (100)	651/685 (95.0)	71/93 (76.3)
Infants partially followed-up (*all follow-up visits not completed*)	59/959 (6.2)	5/147 (3.4)	0/34 (0.0)	33/685 (4.8)	21/93 (22.6)
No of infants lost to follow-up (final outcome unknown)	2/959 (0.2)	0/147 (0.0)	0/34 (0.0)	1/685 (0.1)	1/93 (1.1)
C. **Simplified Treatment given at first level health facility (Subcentre/ Primary Health Centre/ Community Health Centre)**	**N=959 n/m (%)**	**N=147 n/m (%)**	**N=34 n/m (%)**	**N=685 n/m (%)**	**N=93 n/m (%)**
No of infants completed treatment for 7 days	131/959 (13.7)	105/147 (71.4)	16/34 (47.1)	81/685 (11.8)	34/93 (36.6)
D. **Compliance of treatment**	**N=959 n/m (%)**	**N=147 n/m (%)**	**N=34 n/m (%)**	**N=685 n/m (%)**	**N=93 n/m (%)**
**Number of oral amoxicillin doses** 14 Doses	517/959 (53.9)	105/147 (71.4)	31/34 (91.2)	333/685 (48.6)	48/93 (51.6)
10-13 Doses	293/959 (30.6)	41/147 (27.9)	3/34 (8.8)	228/685 (33.3)	21/93 (22.6)
6-9 Doses	10/959 (1.0)	1/147 (0.7)	-	9/685 (1.3)	-
5 or less Doses	17/959 (1.8)	-	-	9/685 (1.3)	8/93 (8.6)
Other oral antibiotics received§	122/959	-	-	106/685	16/93

	(12.7)			(15.5)	(17.2)
**Injection gentamicin received ¶**	**N=812 n/m (%)**	**NA**	**N=34 n/m (%)**	**N=685 n/m (%)**	**N=93 n/m (%)**
7 injections	132/812 (16.3)	NA	16/34 (47.1)	80/685 (11.7)	36/93 (38.7)
5-6 injections	217/812 (26.7)	NA	10/34 (29.4)	192/685 (28.0)	15/93 (16.1)
3-4 injections	90/812 (11.1)	NA	5/34 (14.7)	80/685 (11.7)	5/93 (5.4)
1 -2 injections	73/812 (9.0)	NA	-	52/685 (7.6)	21/93 (22.6)
Other injectable antibiotic (amikacin) received$^{\infty}$	300/812 (36.9)	NA	3/34 (8.8)	281/685 (41.0)	16/93 (17.2)
E. **Outcome for those who accepted simplified treatment (based on respondent perception)**	**N=959 n/m (%)**	**N=147 n/m (%)**	**N=34 n/m (%)**	**N=685 n/m (%)**	**N=93 n/m (%)**
Infants declared as 'Treatment success'	886/959 (92.4)	140/147 (95.2)	34/34 (100)	645/685 (94.2)	67/93 (72.0)
Infants declared as 'Treatment failure'	73/959 (7.6)	7/147 (4.8)	0/34 (0)	40/685 (5.8)	26/93 (28.0)
**Reason for treatment failure**	**N=73 n/m (%)**	**N=7 n/m (%)**	**N=0 n/m (%)**	**N=40 n/m (%)**	**N=26 n/m (%)**
Left treatment	45/73 (62.5)	7/7 (100)	0/0 (0)	30/40 (75)	8/26 (30.8)
Persistence of presenting sign on day 8	1/73 (1.4)	0/7 (0)	0/0 (0)	0/40 (0)	1/26 (3.8)
Deaths	25/73 (34.2)	0/7 (0)	0/0 (0)	09/40 (22.5)	16/26 (61.5)
Outcome unknown	2/73 (2.8)	0/7 (0)	0/0 (0)	01/40 (2.6)	1/26 (3.8)
**F. Outcome of the illness on Day 7 of those who accepted referral and were treated at district hospital**	**N=88 n/m (%)**	**N=1 n/m (%)**	**N=1 n/m (%)**	**N=50 n/m (%)**	**N=36 n/m (%)**
Cured	67/88 (76.1)	1/1 (100)	1/1 (100)	45/50 (90)	20/36 (55.6)
Better	7/88 (8.0)	0/1 (0)	0/1 (0)	02/50 (4)	05/36 (13.9)
Still sick	2/88 (2.3)	0/1 (0)	0/1 (0)	0/50 (0)	02/36 (5.6)
Same	1/88 (1.1)	0/1 (0)	0/1 (0)	0/50 (0)	01/36 (2.8)
Outcome unknown	0/88 (0)	0/1 (0)	0/1 (0)	03/50 (6)	0/36 (0)
Deaths	11/88 (12.5)	0/1 (0)	0/1 (0)	03/50 (6)	08/36 (22)

¶Excluding cases of fast breathing in ages 7-59 days.

* Home remedies, faith healers or no formal treatment

§ Oral amoxicillin was unavailable

∞ Injection gentamicin was unavailable

Of 1302 cases of PSBI, 37 (2.8%) had fast breathing in0-6 days of age young infants, 173 (13.3%) had fast breathing in7-59days of age young infants, 866 (66.5%)had clinical severe infection signs and 226 (20.4%)had critical illness.

Mandatory day 4 follow up was carried in 95.8% (919/959) cases who accepted simplified treatment. Only 13.7% (131/959) received full7 days of WHO recommended simplified treatment course. Of those who accepted simplified treatment at PHC/CHC, 12.7% (122/959) received other oral antibiotics primarily due to non-availability of oral amoxicillin in health facilities; 512/812 (63.1%) who needed injection gentamicin received it, where the rest were treated by injection amikacin, which was available at health facilities. Of the 812 cases of PSBI, who received injectable treatment, injection was given by medial officer in 87.8% (713/812), by ANM in 10.5% (86/812) and by Nurse in 1.6% (13/812) cases. Overall, 92.4% (885/959)PSBI cases who accepted simplified treatment were successfully treated, whereas 2.6% (n=25) died, 9 with signs of clinical severe infection and 16 with critical illness. Of the 88 cases treated at district hospital, 67 (76.1%) were cured, 11 (12.5%) died, 8 with signs of critical illness and 3 with clinical severe infection.

Of the 255 PSBI cases who refused treatment from government facilities, 25.4% (50/197)who sought care from private providers were reported dead on follow-up visits and 79.3% (46/58) who didn’t receive any formal treatment died within 7 days of becoming sick.

During implementation research we faced several challenges and found solutions to many of them in collaboration with the public health authorities **Table 4.**

Table 4: Challenges faced during the implementation of the project

Challenges	Steps taken	Outcome
• HBNC Training had been given to ASHA only at the time of appointment.<br><br>• ASHAs were only weighing the baby and doing verbal inquiry from mothers about the health of the baby. They were not able to carry out all the recommended HBNC activities.<br><br>• HBNC recording form did not have space to record weight, temperature and respiratory rate.	• Re-orientation of ASHAs on above aspects was a regular agenda during cluster meetings with success story sharing.<br><br>• Training and continuous re-orientation of ASHA with hands-on training in field and cluster meetings for:<br>➢ Counting respiratory rate<br>➢ Correct method to record temperature and weight<br><br>• ASHA were requested to document the recordings on the HBNC forms.	• Number of PSBI identification recorded an increase from 1.6% (of live births) in quarter 1 of establishment phase to 9.9% in quarter 1 of implementation phase.<br><br>• Number fast breathing cases identification increased from zero in quarter 1 establishment phase to 14.9% PSBI cases in quarter 1 of implementation phase duration. **(Table 3)**
• ANMs, medical officers (excluding pediatricians) and staff nurses were "hesitant" to administer inj. gentamicin to infants due to fear of adverse events.<br><br>• ANMs also perceived that the community may not be willing for their newborns treated by them, especially administration of injection gentamicin.	• Medical Superintendent motivated them for administration of inj. gentamicin through:<br>➢ Revision of existing government guidelines and role of ANM in simplified treatment of PSBI<br>➢ Addressing safety concerns over administration of gentamicin.<br>➢ Sharing success stories of administration of simplified treatment and pre-referral dose by ANMs.<br><br>• Certificate of appreciation was awarded to ANMs who gave simplified treatment or administered pre-referral dose.<br><br>• A "**job aid**" was provided to ANMs and Medical Officers depicting guidelines to classify and manage a case of PSBI.	• Actual number of cases administered simplified treatment at SC/PHC/CHC increased to even though the proportion remained almost the same. **(Table 3)**<br><br>• Pre-referral drug was administered to 2/8 cases of PSBI in establishment phase which increased to 14/25 cases in implementation phase **(Table 3)**

• No provision for providing oral amoxicillin dispersible tablets and inj. gentamicin to ANMs even though it was available in government supply. One ml syringe was not available in government supply	• Oral amoxicillin dispersible tablets and injection gentamicin and 1 ml syringe with 26-gauge needle were provided through the research funds at the health facilities and ANMs in the establishment phase.	• Injection gentamicin was issued to ANMs by government pharmacists<br>• In Implementation phase government supplied oral amoxicillin dispersible tablets and injection gentamicin and 1 ml syringe with 26-gauge needle.
• Stock out of the HBNC visit recording forms hampered the documentation of the home visits by the ASHAs	• Government authorities were encouraged to regularize the supply of recording forms.	• HBNC forms were supplied to ASHA.
• Low awareness among mothers about neonatal danger signs.<br>• Low acceptance among the community for a referral to the government health facilities	• "**Mother card"** with information about signs of sepsis in newborns was developed and distributed by ASHAs to educate caregivers in village and mothers at the time of discharge after delivery from CHC/ PHC.	• Mother and family were able to identify signs of infection. Most families who identified sickness in their young infants did so in the implementation phase of the program.
• There was loss of critical time in transport between facilities, resulting in delay in treatment.	• The issue was discussed with State Government planners and policy makers to develop a mechanism to obtain information about the availability of a bed at the sick newborn care unit or referral hospitals	• Government endorsed simplified treatment to reduce the burden of cases at tertiary care facilities.<br>• Issue not completely resolved yet and the policy makers are in process of developing information system about availability of beds in NICU to cut short delay during transport
• ASHAs were not able to use the digital watch provided in the HBNC kit. On many occasions they were found non-functional.	• One to one training to each ASHA was given for counting of the respiratory rate by using their mobile phones.	Identification of cases of fast breathing cases by ASHA increased from establishment to implementation phase.
• Posts of ASHA were vacant in some villages.<br>• There are some villages where ASHA are inactive.	• Responsibilities of HBNC visits in such areas were assigned to the ASHAs of the adjacent villages by health administrators.	• Number of first week visit after birth increased from 74.1% in quarter 1of establishment phase to 83.3% in quarter 1of implementation phase.

• ASHAs had to participate in other government campaigns such as for immunization, non-communicable disease etc.	• During Cluster meetings, ASHA were reminded to continue the HBNC program follow-up. TSU staff was directly contacted by ASHAs telephonically when a sick infant was identified.	• There was continuous increase in 1st week visit by ASHA and PSBI case identification despite involvement in multiple programs.<br>• Supportive supervision and hand holding assisted ASHAs to developmulti-tasking abilities.

Abbreviations:

ASHA **Accredited Social Health activist**

HBNC **Home based Newborn Care**

TSU **Technical Support unit**

Training to ASHA was given only at the time of recruitment and since there was no reorientation or supportive supervision, HBNC program was not executed efficiently. Essential equipment such as digital thermometer, weighing scale and digital respiratory rate counter were not functional. Some posts of ASHA were vacant. ANMs did not given oral amoxicillin, injection gentamicin and 1 ml syringes for treat cases of PSBI. There was low awareness to mothers about signs of such illness.

Discussion

In Lucknow public health setting, our data shows that it is feasible to implement the WHO guideline for PSBI management when referral is not feasible. First, the overall coverage rate in a birth cohort of 25028 was 53%, which is about half the expected, but we achieved a reasonably high coverage of 85% of PSBI cases in the implementation phase. ~~However,.~~Second, ASHAs were able to identify a large number of sick young infants under a strengthened HBNC. With training, supportive supervision and mentoring, the rate of identification of PSBI cases by ASHAs in the community increased from about 2% in the

beginning to 8-10% at the end, which was similar to reported elsewhere [5]. Third, young infants 7-59 days of age with only fast breathing were successfully and safely treated with oral amoxicillin at PHC/CHC level. Fourth, young infants with signs of clinical severe infection whose parents refused referral advice to a hospital were successfully managed at an outpatient level with a combination of oral amoxicillin and intramuscular gentamicin with low case fatality rates. These high rates of treatment success and low case fatality are similar to data reported from other parts of Asia and Africa [13, 16, 17, 18, 19].

High acceptance of simplified treatment at the government facilities showed the confidence of caregivers in them. This high acceptance is similar to that reported from both Asian and African sites, which have documented high refusal rates to acceptance of referral advice [13, 16, 17, 18, 19]. Several reasons for not accepting referral advice have been reported, such as distance to the hospital, cost of travel and treatment, concern about the quality of care or attitude of the health workers, lack of permission from family members, religious and cultural beliefs, and issues with lack of child care and other logistical problems [30, 31]. Unless these issues are addressed there will continue to be a large proportion refusing referral advice, especially in rural and less educated populations.

In the simplified treatment regimen, administration of daily injection gentamicin for 7 days was a perceived challenge and over 40% received at least up to 5 doses. Even though, compliance to all 7 doses was low, still very good treatment success was observed, supporting the WHO guideline that even two injections of gentamicin were effective [15].

The success rate of simplified treatment at PHC/CHC was higher and case fatality lower compared to those who were treated at a district hospital. This could be due to the fact that parents would opt for a higher facility for treatment if they perceived their infant to have more severe illness, or a delay caused by inappropriate care seeking and treatment elsewhere

or difficulty in getting transport to the hospital and sometimes not receiving pre-referral antibiotic treatment. But the most worrisome finding is high case fatality of those sick young infants whose parents refused formal treatment at all or sought private treatment, which could have been from inappropriate treatment provided. If the government is serious in addressing high neonatal mortality in the country then this needs to be addressed through community engagement strategies and improving quality of care in private sector. Empowering families through community activities in rural areas to create awareness to identify illness and seek appropriate care promptly is key to early management of sick young infants.

The main strength of our study was that this implementation research was executed within the existing Government health system. Capacity strengthening of government functionaries and infra-structure improvement was carried out within a short period of nine months. Recognition of cases of PSBI by grass-root workers, ASHAs, almost tripled. Initial perception that giving injection gentamicin by the ANM in simplified treatment to young infants staying at home would be difficult proved incorrect. High coverage of simplified treatment was achieved by training, supportive supervision of physicians in the public health system who also acted as agents of change by setting an example. However, certain logistic weaknesses persisted despite close engagement of TSU with the health system. Most important was lack of medicines at the point of care. This resulted in use of alternate oral and injectable antibiotics. Simplified treatment was offered only when caregivers of cases of PSBI refused referral. Information about success rate of simplified treatment was not given to the parents. This resulted in caregivers still opting for treatment from private providers, who could have been unqualified or in the worst scenario not accepting any treatment. When simplified treatment of PSBI is rolled out at the state and national level, a strong communication component has to be integrated for community buy-in which is essential for successful reduction of neonatal mortality rate.

At the end of the implementation phase public health trainers also trained other doctors, nurses, ANMs and ASHAs in the remaining four blocks of Lucknow district that were not in the implementation phase. The government was requested to fast track procurement of oral amoxicillin and injection gentamicin. ANMs were issued gentamicin injection vials for use in cases of PSBI in the community.

During the establishment phase, only 1.6% cases of PSBI were identified as compared to 8.7% in the implementation phase, which was nearer to the PSBI rate of 7·6% (95% CI 6·1–9·2%) from a meta-analysis[4]. Thus prior to the implementation research, almost 3/4th cases of PSBI were missed. This would have contributed to high neonatal mortality rate in Uttar Pradesh.

Our findings have shown that a substantial number of neonatal deaths can be averted through this simple intervention for management of PSBI cases. Our experience and that from other sites in India will hopefully give confidence to the government to seriously consider scaling up implementation of PSBI treatment guidelines through the country in areas where referral is not feasible. However, it will require continuous skills upgradation, supportive supervision and motivation of grass-root workers by their supervisors and physicians and ensuring continuous availability of supplies. A national and state level commitment is needed to reduce neonatal mortality through strengthening HBNC and effective implementation of simplified treatment of PSBI when referral is not feasible. Fortunately, the government of India already has issued a guideline [14, 20] but it needs more than just a document. Role of TSU in this implementation research was critical in achieving these positive results. When scaled up it is not essential to have a TSU, but technical support from experts will be needed to address the confidence issues in health workers and health system challenges to achieve high coverage of treatment with high quality. The challenges faced during this study were

addressed in a good working partnership between the TSU and the district managers and implementers.

We compiled 30 success stories of simplified treatment in local Hindi language. The state government obtained 1000 copies of these for distribution across the state to motivate health care workers. During the same time, government of India released national guidelines endorsing simplified treatment of PSBI. [20]. The state government now has first-hand experience in rolling out simplified treatment under program setting in the pilot implementation area and with endorsement from central government, the state government is planning to expand simplified treatment for PSBI to remaining 74 districts of Uttar Pradesh.

Conclusion

Simplified treatment for PSBI is feasible in public program settings in northern India with good cure rates. It required system strengthening and supportive supervision. There is a need to rollout this program on priority basis to reduce neonatal mortality.

Acknowledgements

We thank the Ministry of Health and Family Welfare for giving us the permission and Late Dr. Anil Verma, General Manager, Child Health for support to conduct this implementation research. We also extend our gratitude to all the medical superintendents, medical officers, workers of the community process department, community health workers and parents of young infants of the rural blocks of Mall, Kakori, Gosaiganj and Sarojini nagar for their participation in the study.

References

1. Office of Registrar General and Census Commissioner, India. Sample Registration System Statistical Report [Internet]. 2017. [cited 26 december 2019]. http://www.censusindia. gov.in/vital_statistics/ SRS_Reports_2017.html

2. Fadel SA, Rasaily R, Awasthi S, Begum R, Black RE, Gelband H, et al. Million Death Study. Changes in cause-specific neonatal and 1-59-month child mortality in India from 2000 to 2015: a nationally representative survey. The Lancet. 2017 Oct 28;390(10106):1972-1980.

3. Wang ME, Patel AB, Hansen NI, Arlington L, Prakash A,Hibberd PI.Risk factors for possible serious bacterial infection in a rural cohort of young infants in central India. BMC Public Health. 2016; 16 (1): 1097.

4. Seale AC, Blencowe H, Manu AA, Nair H, Bahl R, Qazi SA, et al. Estimates of possible severe bacterial infection in neonates in sub-Saharan Africa, south Asia, and Latin America for 2012: a systematic review and meta-analysis. Lancet Infect Dis. 2014 Aug 1;14(8):731-41.

5. Seale AC, Blencowe H, Zaidi A, Ganatra H, Syed S, Engmann C, et al. Neonatal severe bacterial infection impairment estimates in South Asia, sub-Saharan Africa, and Latin America for 2010. Pediatr Res. 2013 Dec 20;74(S1):73-85.

6. World Health Organization. WHO recommendations on postnatal care of the mother and newborn [Internet].2013. [cited 14 february 2020]. https://www.who.int/maternal_child_ adolescent/documents / postnatal-care-recommendations/en/

7. Ministry of Health and Family Welfare. Government of India. Revised Home Based Newborn Care. Operational Guidelines[Internet]. 2014. [cited 14february 2020]. http://tripuranrhm.gov.in/ Guidlines/2205201401.pdf

8. World Health Organization. Pocket book of hospital care for children: guidelines for the management of common childhood illnesses. Geneva, World Health Organization [Internet]. 2013.[cited 14 february 2020]. https://www.who.int/maternal_ child_adolescent/documents /child_hospital_care/en/

9. World Health Organization. Integrated Management of Childhood Illness (IMCI) Chart Booklet[Internet]. 2014.[cited 14 february 2020]. https://www.who.int/maternal_ child_adolescent/documents / IMCI_chartbooklet/en/

10. Baqui AH, Arifeen SE, Williams EK, Ahmed S, Mannan I, Rahman SM, et al. Effectiveness of home-based management of newborn infections by community health workers in rural Bangladesh. Pediatr Infect Dis J. 2009 Apr;28(4):304-10.

11. Bang AT, Bang RA, Baitule SB, Reddy MH, Deshmukh MD. Effect of home-based neonatal care and management of sepsis on neonatal mortality: field trial in rural India. The Lancet. 1999 Dec 4;354(9194):1955-61.

12. Khanal S, Sharma J, Singh V GC, Dawson P, Houston R, Khadka N, et al. Community Health Workers Can Identify and Manage Possible Infections in Neonates and Young Infants: MINI-A Model from Nepal. J Health PopulNutr. 2011 Jun;29(3):255.

13. Zaidi AK, Tikmani SS, Warraich HJ, Darmstadt GL, Bhutta ZA, Sultana S, et al. Community-based Treatment of Serious Bacterial Infections in Newborns and Young Infants. Pediatr Infect Dis J. 2012 Jul 1;31(7):667-72.

14. Ministry of Health and Family Welfare. Government of India. Use of gentamicin by ANMs for management of sepsis in young infants under specific situations- Operational Guidelines[Internet]. 2014. [cited 24 february 2020]. https://www.healthynewbornnetwork.org /hnn-content/uploads / Operational-Guidelines_India.pdf

15. World Health Organization. Guideline - Managing possible serious bacterial infection in young infants when referral is not feasible. [cited 24 february 2020]. https://apps.who.int/iris/bitstream /handle/10665/181426/9789241509268_eng.pdf?sequence=1

16. Tshefu A, Lokangaka A, Ngaima S, Engmann C, Esamai F, Gisore P *et al.*Oral amoxicillin compared with injectable procaine benzylpenicillin plus gentamicin for treatment of neonates and young infants with fast breathing when referral is not possible: a randomized, open-label, equivalence trial. The Lancet. 2015 May 2;385(9979):1758-66.

17. Tshefu A, Lokangaka A, Ngaima S, Engmann C, Esamai F, Gisore P *et al.*Simplified antibiotic regimens compared with injectable procaine benzylpenicillin plus gentamicin for treatment of neonates and young infants with clinical signs of possible serious bacterial infection when referral is not possible: a randomized, open-label, equivalence trial. The Lancet. 2015 April 1;385(9979):1767-76.

18. Mir F, Nisar I, S Tikmani SS, Baloch B, Shakoor S, Jehan F et al. Simplified antibiotic regimens for treatment of clinical severe infection in the outpatient setting when referral is not possible for young infants in Pakistan (Simplified Antibiotic Therapy Trial [SATT]): a randomised, open-label, equivalence trial. Lancet Glob Health. 2017 Feb 1;5(2):e177-85.

19. Baqui AH, Saha SK, Ahmed AS, Shahidullah M, Quasem I, Roth DE, et al. Safety and efficacy of alternative antibiotic regimens compared with 7 day injectable procaine benzylpenicillin and gentamicin for outpatient treatment of neonates and young infants with clinical signs of severe infection when referral is not possible: a randomised, open-label, equivalence trial. Lancet Glob Health. 2015 May 1;3(5):e279-87.

20. National Health Mission. Government of India. Addendum to Operational Guidelines on Use of Gentamicin by ANMs for Management of Sepsis in Young Infants under Specific Situations [Internet]. 2018. [cited 14 february 2020].https://nhm.gov.in/New_Updates_2018 /Om_and_orders/rmncha/child_health/Addendum.pdf

21. Government of India. Census of India 2011. Directorate of Census Operations, Uttar Pradesh[Internet]. 2011. [cited 14 february 2020].https://www.census2011. co.in/census / metropolitan/82-lucknow.html

22. Government of India. District Census Handbook, Census of India 2011.Directorate of Census Operations, Uttar Pradesh [Internet].2014. [cited 24 february 2020].http:// censusindia.gov.in/2011census/dchb/DCHB_A/09/0926_PART_A_DCHB_LUCKNOW. pdf

23. National Health Mission. Ministry of health & Family welfare, government of India. About Accredited Social Health Activist (ASHA) [Internet]. [cited 14 february 2020].https://nhm.gov.in / index1.php?lang=1&level=1&sublinkid=150&lid=226

24. Government of India. Indian Public Health Standards (IPHS). Guidelines for Sub-Centres (Revised 2012)[Internet]. [cited 16february 2020].https://nhm.gov.in/ images/pdf/guidelines/iphs/ iphs-revised-guidlines-2012/sub-centers.pdf

25. Government of India. Indian Public Health Standards (IPHS). Guidelines for Primary Health Centres (Revised 2012)[Internet]. [cited 16 february 2020].https:// nhm.gov.in/images/pdf/ guidelines/iphs/iphs-revised-guidlines-2012/primay-health-centres.pdf

26. Government of India. Indian Public Health Standards (IPHS). Guidelines for Community Health Centres (Revised 2012)[Internet]. [cited 24february 2020].https://nhm.gov.in/ images/pdf/guidelines/iphs/iphs-revised-guidlines-2012/ community-health-centres.pdf

27. World Health Organization. Training for mid-level managers (MLM) module 4: Supportive supervision. World Health Organization's Publications[Internet]. 2008. [cited 24february 2020].https://www.who.int/immunization/documents/mlm/en/

28. RedCap Software. REDCap Vanderbilt 2006. [cited 24february 2020].https://www.project-redcap.org/ software/

29. SPSS Inc. Released 2009. SPSS for Windows [software], Version 18·0. Chicago, SPSS Inc. [cited 24february 2020].www.spss.com

30. Kozuki N, Guenther T, Vaz L, Moran A, Soofi SB, Kayemba CN, et al. A systematic review of community-to-facility neonatal referral completion rates in Africa and Asia. BMC Public Health. 2015 Dec;15(1):989.

31. Owais A, Sultana S, Stein AD, Bashir NH, Awaldad R, Zaidi AK. Why do families of sick newborns accept hospital care? A community-based cohort study in Karachi, Pakistan. J Perinatol. 2011 Sep;31(9):586-92.

Supporting Information

S1 Table: The public health infrastructure available in Lucknow district and in 4 Implementation Blocks

S2 Table: Actions taken over responses of family of sick infant in community/ PHC/CHC

S3 Table: Initial training in four Intervention blocks

S4 Table: Re-orientation training of ASHAs and ANMs in the intervention blocks (Nov 2017-Feb 2019)

 Click here to download Figure Fig1.tif

Figure 1: Places of Identification and Management of cases of Possible Serious Bacterial Infections

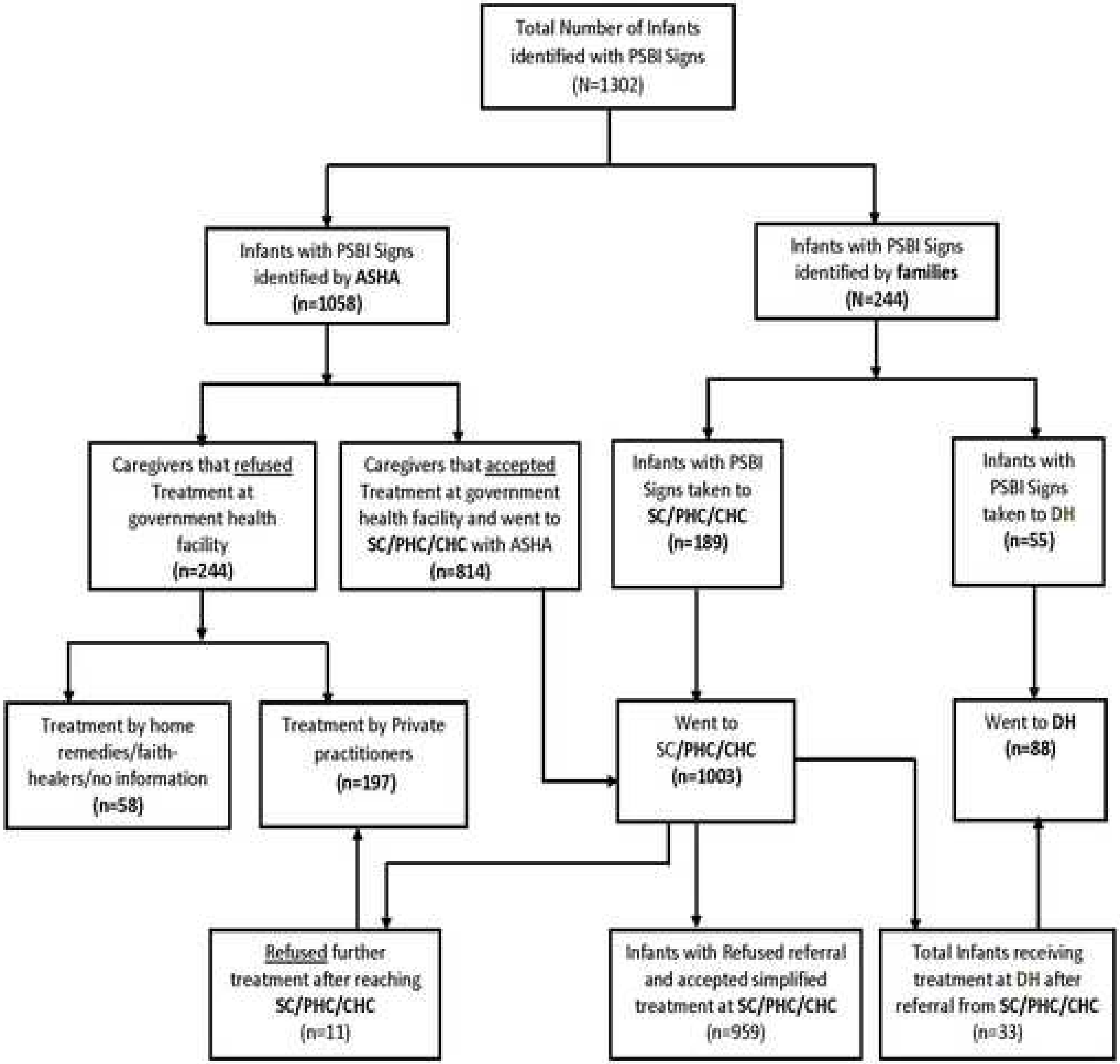

SC = Sub Centre, PHC = Primary Health Centre, CHC = Community Health Centre, DH = District Hospital; FB=Fast Breathing

Supporting Information 1

Click here to access/download
Supporting Information
S1 Table.docx

Click here to access/download
Supporting Information
S2 Table.docx

Click here to access/download
Supporting Information
S3 Table.docx

Click here to access/download
Supporting Information
S4 Table.docx

Supplementary Table 1: The public health infrastructure available in Lucknow district and in 4 Implementation Blocks

Health Units	Lucknow District	4 Implementation blocks
Community Health Centers (CHC)	09	04
Primary Health Centers (PHC)	33	16
Sub-centers	321	155
Pediatricians at CHCs	9	04
Medical Officers at PHCs	35	18
Auxillary Nurse Midwives	344	176
Accredited Social Activists (ASHAs)	1486	750
ASHA Supervisors, Staff nurses/Lady Health Visitor(Supervisory Staff)	170	95

Supplementary Table 2: **Actions taken over responses of family of sick infant in community/ PHC/CHC**

If the family accepts referral for hospitalization-	• Arrangement of transport provided free under *JSSK**<br>• ASHA may accompany baby to the nearest CHC/ SNCUs/ tertiary care hospital
If the family refuses referral for hospitalization BUT ready to visit health facility	• ASHA accompanied the baby to the nearest PHC /CHC/SC for diagnosis and treatment/ medical advice<br>• MO/SN/ANM assesses the young infant<br>• Counselled parents for urgent referral to CHC/ DH/ TCC (Tertiary care centre)<br>• Arranged transport and explained whom to contact at the referral facility.<br>• Provided Pre-referral dose – 1st dose of each antibiotic
If the family refuses to accept hospital referral and prefers home treatment	• ASHA accompanied the PSBI case to the PHC/ANM for confirmation<br>• ANM/ TSU assessed the sick Young Infant, Proposes simplified treatment at SC/home<br>• Family visits SC/PHC/CHC daily for simplified treatment dose<br>• ASHA followed up the YI daily at home till Tt completed
If family refuses to attend even the first level health facility	• ASHA contacted the ANM and explained the situation.<br>• Concerned ANM visited the sick YI at home and informed MO<br>• ANM followed the Sick YI between 10-14 days for relapse or a new morbidity.<br>• ASHA visited the house daily to observe the child till 14 days

***JSSK*-** *Janani Shishu Suraksha Karyakaram* scheme provides free referral transport for new-born.

Supplementary Table 3: Initial training in four Intervention blocks

Type of participants [Timing of training]	Numberof Batches	Number of participants [Types]	Duration (days)	Methods	Content of training	Training Material
**Training of Master trainers [November 2016]**	01	03 [national level training of project investigators]	03	Class room training - cases studies and videos.<br>Clinical practice sessions in the wards	Assessment, classification and treatment of sick young infant for very severe disease (PSBI), Pneumonia, local infection, feeding problem, and low birth weight	1. Facilitator guidelines- *Management of the sick young infant of age up to 02 months*<br>2. Participant manual- *Management of the sick young infant of age up to 02 months*<br>3. Chart booklet<br>4. Photo booklet-Photograph for physicians-IMNCI (*WHO-UNICEF*)
**Training of State Level Trainers [April 2017]**	01	24 (Pediatricians/MOs posted at CHCs, DH, and SNCUs)	03			
**Training of Medical Officers [May, Aug, Sept 2017]**	04	11 [Medical officers of PHCs at their respective CHCs**]**	03	Classroom training using cases studies and videos and practical training at nearby SNCUs		1. Participant manual<br>2. Chart booklet<br>3. Photo booklet-Photograph for physicians-IMNCI (*WHO-UNICEF*)
**Training of ANMs and staff nurses at their respective CHCs [May 2017]**	04	137 (20-30 participants/batch)	03		Same as for medical doctors plus skill development for giving Inj Gentamicin	1. Participant manual<br>2. Chart booklet<br>3. Photo booklet<br>4. Photograph for physicians-IMNCI (*WHO-UNICEF*
**Training of community Health workers(ASHA) and their supervisors [June 2017]**	14	700 (45-55/batch)	01	Classroom Training using videos	Identification of danger signs in sick young infants and referral	**Chart booklet** –Management of the sick young infant (translated in Hindi)<br>**Picture booklet**

Supplementary Table 4: Re-orientation training of ASHAs and ANMs in the intervention blocks (Nov 2017-Feb 2019)

**Meetings**	**Participants**	**Number of meetings**	**Activities are undertaken by Project team**	**Support from government officials**
Monthly Cluster* meetings at Community Health centers	CHWs (ASHA) and ASHA *Sangini*	215 meetings (50-60 meetings per CHC)	• Reorientation of ASHAs on the identification of danger signs in young infants with focus on the seven of PSBI in a young infant<br>• Handholding of ASHA to count RR and correct use of thermometer in a young infant<br>• Discussion on problems faced by ASHA in the documentation of HBNC visit and handholding for maintenance of records<br>• Experience sharing by ASHAs who identified a young infant with danger signs and successfully referred the case to a health facility	• Support was given by Health Education Officer (HEO) of the respective CHC to the project team in reorientation and handholding of ASHAs<br>• Some Cluster meetings were also attended by the Medical superintendent of the CHC.
Weekly meetings of ANMs at CHCs	ANMs	175 meetings (40-50 meetings per CHCs)	• Reorientation of ANMs on assessment, classification and pre-referral treatment of sick young infant.<br>• Identification of seven PSBI signs in a young infant<br>• Motivation to administer Pre referral dose(PRD) before referral of a PSBI case to an Health Facility<br>• Revision of Simplified treatment (inj. Gentamicin and distab/syp. Amoxicillin) using Job aids<br>• Felicitation of ANMs by awarding "Appreciation Certificate"	Support by HEOs and Medical Superintendent in reorienting ANMs on assessment of Sick young infants and motivating them to administer PRD before referring sick young infant to the HF

*Cluster is a group of 20 ASHAs headed by a supervisor known as ASHA supervisor. There are approximately 8-10 clusters in each block.
