# Supplementary material for: Identification and management of young infants with possible serious bacterial infection where referral was not feasible in rural Lucknow district of Uttar Pradesh, India: An implementation research
Source: PLoS One. 2020 Jun 4;15(6):e0234212. doi: 10.1371/journal.pone.0234212 (PMC7272098; doi:10.1371/journal.pone.0234212)
Supplement: Attachment — Submitted filename: Response to Reviewers Comments_19.55.2020_SA.docx [file pone.0234212.s006.docx]

*Title: Identification and management of young infants with possible serious bacterial infection where referral was not feasible in rural Lucknow district of Uttar Pradesh, India: An implementation research.*

**Reference:PONE-D-20-05531**

**RESPONSE TO REVIEWER COMMENTS**

**Reviewer #1: The manuscript addresses an important implementation research undertaken in one of the challenging areas of India.**

| **Serial No.** | **Reviewer #1 Comment** | **Response** | **Line No.** | **Page No.** |
| --- | --- | --- | --- | --- |
| 1 | The manuscript primarily presents the quantitative findings from the study. The qualitative component of the study is not presented adequately. | We have added relevant findings of qualitative research. | 299-340;  364-389 | 14-16;  19-20 |
| 2 | The findings from Baseline, Midline survey have not been presented adequately. The findings of the interview and data collection from mothers have not been presented adequately. | We have added relevant finding in the manuscript and also added **Table 1** and **Table2**. | 298-389 | 14-20 |
| 3 | The findings presented in the tables vary in terms of the differ in periods- somewhere the establishment phase and implementation phase have been combined, while at other places they are presented separately. A consistent pattern shall be useful. | **Table 1** gives basic demographic details, **Table 2** gives change in knowledge of mother and ASHAs about danger signs and **Table 3** shows performance indicators during the initial “establishment phase” and the later “Implementation phase” whereas  **Table 4** shows identification and classification in young infants with Possible Serious Bacterial Infection.  **Table 5** gives compliance and outcome of cases of Possible Serious Bacterial Infection who were treated with Simplified treatment and who accepted referral and were treated at district hospital. |  |  |
| 4 | Results section: The findings may start with the findings of the PSBI management and the variations or potential reasons may be explained from the qualitative and process observations. | We have modified accordingly. We have begun with findings of qualitative results as suggested. | 299-340;  364-389 | 14-16;  19-20 |
| 5 | The performance of the ANMs (10.5%) for administration of gentamicin should have been furthered explained with the trend across the implementation period. As this is important and relevant from other parts of India and other LMICs. | We have elaborated as “ANMs administered injectable treatment to 44 cases in establishment and 42 in implementation phase. This treatment was administered by 34.7 %( 41/118) ANMs.” | 446-448 | 26 |
| 6 | Overall the detection of PSBI cases was lower than anticipation. Possible explanations for this may have to be explored. | During the start of this study we noticed that there was no identification of cases of fast breathing as the ASHA/ ANM were not counting the respiratory rate. They were trained in counting the RR during this research and detection of cases improved.  We have now written this in the discussion “Improved detection of cases of PSBI in the implementation phase can be attributed to skills upgradation, supportive supervision and availability of complete HBNC kit of ASHA workers.” | 537-539 | 32 |
| 7 | The variations across the 4 blocks and facilities, was there noticeable differences in the performance - detection of PSBI cases, management and compliance. If any variation observed, what were the  possible reasons, as these learning shall inform the future scaling up. | There was no statistically significant difference in case management across the 4 blocks and individual facilities. |  |  |
| 8 | Supplementary Table 2: The language need to be appropriate and consistent. The abbreviations need to be expanded below. | Language corrected and Abbreviation expanded |  |  |

Reviewer #2: The paper provides outcomes of an operational research on identification and management of possible serious bacterial infection conducted in Uttar Pradesh, India in a community based setting where referral is not possible. The study was well planned and executed and aligned with the government program for addressing infant mortality. The treatment protocol followed the WHO guidelines and guidelines issued by Ministry of Health in India. The article can be published in the journal after the authors make following revisions provided below.

| **Serial No.** | **Reviewer #2 Comment** | **Response** | **Line No.** | **Page No.** |
| --- | --- | --- | --- | --- |
| **Introduction** | | | | |
| 9 | Line 40: This line is not clear “mortality rate in India is 33 versus 41 per 1000 live births’ what is India’s IMR, UP IMR, etc. | Updated as advise now we have written as  “In India, the Infant mortality rate per 1000 live births is 33 as compared to 41 in Uttar Pradesh, the most populous state.” | 39-42 | 4 |
| 10 | The introduction talks about the HBNC program, how is it done, ASHA, etc. but it is not clear from the write-up why do they want to do this study. Are there studies already available or no data or what are the lacunae in the existing evidence? This is totally missing: the rationale of doing this study? | The rationale has been mentioned in the manuscript as below:  “Despite the HBNC program going on from 2011 there is a high neonatal mortality rate in India, and 41 % of deaths are due to pneumonia and sepsis [7]. Therefore, the rationale and primary objective of this implementation research was to evaluate acceptability, effectiveness and scalability of simplified treatment regimen to cases of PSBI when referral is not accepted in a HBNC program setting in Uttar Pradesh.” | 87-91 | 6 |
| 11 | The authors seem confused whether it is operational research or implementation research. | This is implementation research and the manuscript has been updated accordingly. |  |  |
| 12 | Line 69-71: the authors suddenly jumped from operational research to implementation research. If the  authors want to describe about the implementation, they need to write about it separately (a separate para in the introduction section) because the operational study was done in UP and IR was done in  other states. | As discussed in point 11, this is implementation research. |  |  |
| **Methodology** | | | | |
| 13 | Line 76: This is not clear. Whose birth rate are we talking about? Uttar Pradesh or Lucknow‘ has a crude birth rate of Uttar Pradesh is 25.9 per 1000 population’. | The sentence has been updated to “Uttar Pradesh has a crude birth rate of 25.9 per 1000 population [1].” | 99 | 6 |
| 14 | Line 116: the reference has been wrongly put. ‘at SC. [24]They’ | Location of the reference number has been correctly placed as  “ANMs are responsible for routine immunization at SC/PHC/CHC and administration of certain drugs/ treatment approved to community at SC [21].” | 82-83 | 5 |
| 15 | Line 82-84: the program and its activities have been detailed excessively. This is too much for the reader to absorb. Can the additional details of the program be explained separately or in the introduction. Because in the methodology section, it’s the methodology of the study that should come and not the program. | Modified as suggested. We have added it in introduction. | 71-91 | 5-6 |
| 16 | What mechanisms were followed by ASHAs for not missing out any case for investigation, as case  identifications were done at the community level. | 1. ASHAs made their contact numbers available with the family. She also explained to them signs of illness. If a young infant had any danger signs, the family contacted ASHA between the scheduled visits. 2. There were government supervisors who monitored the working of ASHA to ensure that PSBI cases were not missed. |  |  |
| 17 | Apart from training and ongoing orientation, how it was ensured that investigation conducted by ASHAs in the community were accurate and there were no ‘false negative’ cases? Also, how many  cases identified by ASHAs were ‘false positive’? | We do not have data on these cases as these were not a part of the objectives of the implementation research. |  |  |
| 18 | What strategies were adopted to improve treatment compliance and motivating parents to come for regular treatment of their child as only around 54% of children took all 14 doses of oral amoxicillin and only 16.3% of children received all 7 doses of Gentamycin injection? | We had written this in the original manuscript as “ASHA visited such cases almost daily to ensure adherence to treatment” and this is also written in revised manuscript.  Now we have added to manuscript that “Since injectable gentamicin was not available in the government hospitals all the time, it was replaced by injection amikacin. Hence compliance to all seven injections gentamicin was low (132/812, 16.3%).” | 149-150  426-428 | 8  24 |
| **Results** | | | | |
| 19 | Can table 3 be reduced or broken down into 2 different tables? It is too long and difficult to read. | This table has been broken into two tables and now numbers as table number 4 and 5. | 418-421  429-435 | 22-24  24-26 |
| 20 | It would be good if comparative findings of two cross-sectional surveys-baseline and midline are  presented to present change in the knowledge and skills of ASHAs and ANMs in terms of identification and management of PSBI as this is important before transition into the implementation phase. | As per advice, table numbers 1 and 2 have been added. | 345-346  352-353 | 17  18 |
| 21 | Reasons need to be provided for infants (6.4%) who did not complete the mandatory follow ups on Day 4 and Day 8. | The possible reasons were migration of families. This has been added as “Day 4 and 8 follow-up was done in 93.6 % (898/959) cases of PSBI on simplified treatment. Those lost to follow up were largely due to migration of families.” | 415-417 | 22 |
| 22 | Also, reasons need to be explained for less compliance. | We have explained as “Reasons for low compliance to simplified treatment could be either family stopped treatment due to improvement in sickness or health centre being far away or family opting for treatment elsewhere.” | 423-426 | 24 |
| **Discussion** | | | | |
| 23 | To assist policy decision, as a future research, authors can consider conducting the ‘cost effectiveness analysis’ of identification and management of young infants with PSBI, where referral was not feasible. | This has been done and will be given in detail as a separate manuscript. |  |  |

**Other specific comments are in the text as comments/edits.**

| **Serial No.** | **Reviewer #1 Comment** | **Response** | **Line No.** | **Page No.** |
| --- | --- | --- | --- | --- |
| 24 | Please specify the two sub categories. | Sub Categories has been provided as – “WHO, in 2015, published the guideline for management of PSBI in young infants when referral is not feasible [15] based on evidence from several countries [13, 16, 17, 18, 19], which recommended a simplified treatment regimen with oral amoxicillin plus injection gentamicin for treatment of clinical severe infection and severe pneumonia.” | 65-66 | 5 |
| 25 | It may be good to clarify that this is part of a multisite study conducted at four sites across four states in India and the current report focuses on the findings from one site in Uttar Pradesh. | As per recommendation, we have added statement “. This implementation research was part of a multisite study conducted at four sites one each in four states in India. The current manuscript focuses on the findings from Lucknow site in the state of Uttar Pradesh. The other three other states where this study was conducted were- Haryana, Himachal Pradesh and Maharashtra.” | 91-95 | 6 |
| 26 | Specify the year | As per advice year has been added as “Census of 2011”. | 101 | 6 |
| 27 | A range should be better | We have updated the statement- ”one Primary Health Center (PHC) covering a population of 30,000 to 50,000” | 112-114 | 7 |
| 28 | Should add the PSBI cases, treatment given and referrals made | The ANMs did not maintain any record reported PSBI cases anywhere. |  |  |
| 29 | After what period? | We have updated it as “on day 8 of follow up” | 151-154 | 8 |
| 30 | Period/month and year may be useful | Information provided “November 2016” | 202-203 | 10 |
| 31 | Please indicate the number of participants targeted under each category. | The target population included all the young infants, aged 0- 59 days on the day of visit to the specific listed village. |  |  |
| 32 | or household | The unit of survey was all the infants of the village under infants, aged 0- 59 days. |  |  |
| 33 | Was the reason for non-acceptance of referral documented? | The possible reasons for not accepting referral were healthcare centre being far away and lack of funds. |  |  |
| 34 | It shall be better to use the same terminology 2 months or 59 days. | The manuscript has been updated as advised and changed from ‘2 months’ to “infants, aged 0-59 days” |  |  |
| 35 | Findings from the baseline, midline is not presented clearly.  findings from the mothers not presented and the changes in the knowledge status not reflected. | The comparative findings of baseline and midline are shown in Table 1 and 2. | 298-389 | 14-20 |
| 36 | Was a kit with 7 ASHAs not available? | ASHA kits were given to all ASHA after completion of training. The thermometers were often went out of order however, they were replaced by the administration |  |  |
| 37 | This should be the first paragraph of Results section. | As per advice, this paragraph has been moved to the beginning of results section. | 293 -297 | 14 |
| 38 | The project was implemented in two phases - June 2017 to Feb 2018 and Mar 2018 -Feb 2019. It appears that the phases had different intensity and implementation rigor. Thus it shall be better to represent these two phases separately and then a pooled observation for better clarity- as given in Table 2. | The project was implemented in two phases – Establishment phase (June17 –Feburary18) and Implementation phase (March 18- February 19). Observations regarding home based new born care visit, identification and management of PSBI cases are now presented in **Table3**, phase wise. | 399-402 | 21 |
| 39 | Were these injections given by few ANMs only. A number of ANMs who administered shall be useful. | The manuscript has been updated as “ANMs administered injectable treatment to 44 cases in establishment and 42 in implementation phase. This treatment was administered by 34.7 %( 41/118) ANMs” | 446-448 | 26 |
| 40 | As there is information about the population, live births, is it possible to estimate the change in the NMR/Young infant mortality rate with PSBI compared to the pre-intervention period. | Since we do not have data about non-PSBI related death, we will not be able to estimate the change in NMR/Young infant mortality rate. This was also not the part of the study. |  |  |
| 41 | It may be useful to present the number of ANMs administered the injections. | Updated as “Out of these, 02 pre-referral drugs were given by ANM.” in Table 6. | 459-463 | 27-29 |
| 42 | Unbold the abbreviations | Updated as advised. | 460-463 | 29 |
| 43 | Which coverage rate? | In sentence “First, the overall coverage rate in a birth cohort of 24448 was 53%”, 53% coverage rate shows the combined coverage rate of establishment and implementation phase. | 472-475 | 29 |
| 44 | Adding the % would be useful | Updated as advised. Now it has been written as “ Third, 95.2 % (140/147) of young infants (age 7-59 days) with fast breathing were successfully and safely treated with oral amoxicillin at PHC/CHC level” | 478-480 | 29 |
| 45 | Adding the % would be useful | Updated as advised. Now it has been written as “Fourth, 94.2% (645/685) of young infants with signs of clinical severe infection whose parents refused referral advice to a hospital were successfully managed at an outpatient level with a combination of oral amoxicillin and intramuscular gentamicin with low case fatality rates.” | 480-483 | 29 |
| 46 | On whom? The sentence may be rewritten. | As per advice this has been updated as:  “High acceptance of simplified treatment at the government facilities showed the confidence of caregivers in health care providers ” | 486-487 | 30 |
| 47 | How do the factors/reasons identified in the current study resemble or differ from those reported in the literature? | No new changes have been identified. |  |  |
| 48 | % may be better | Updated as advised “ Even though, compliance to all 7 doses was low, still very good treatment success of 92.4% was observed, supporting the WHO guideline that even two injections of gentamicin were effective [15]. ” | 496-498 | 30 |
| 49 | Language (tense) and abbreviations to be addressed. | As per advised language corrected and all abbreviations are mentioned in **S2 Table**. |  |  |
| 50 | RedCap related:  Using mobile phones or Tablets | We used paper-based data collection which was later verified and entered in Redcap software. |  |  |
